# Retrorectal adenocarcinoma arising from tailgut cysts: a rare case report

**DOI:** 10.1186/s12893-019-0639-9

**Published:** 2019-11-27

**Authors:** Wei Li, Jian Li, Ke Yu, Kai Zhang, Jiannan Li

**Affiliations:** 1 Department of General Surgery, The Second Hospital of Jilin University, 218 Ziqiang Street, Nanguan District, Changchun, 130041 Jilin Province China; 2 Department of Pathology, The Second Hospital of Jilin University, Changchun, Jilin China; 3 Operating Theater and Department of Anesthesiology, The Second Hospital of Jilin University, Changchun, Jilin China

**Keywords:** Tailgut cysts, Retrorectal adenocarcinoma

## Abstract

**Background:**

Tailgut cysts arise from the remnants of the tailgut during the embryonic period. Although malignant transition of tailgut cysts is very rarely observed in the clinic, this congenital condition should be carefully monitored for early diagnosis and appropriate treatment, especially when the tailgut cysts are malignant.

**Case presentation:**

Here, we report the case of a 33-year-old man with retrorectal adenocarcinoma originating from the tailgut cysts. Magnetic resonance imaging (MRI) showed many cystic masses in the posterior rectal space, the largest of which was approximately 100 mm × 59 mm × 53 mm in size and compressed the rectum. The patient underwent surgical resection of the masses located in the retrorectal and anterior sacral spaces. Histological and immunohistological examinations confirmed adenocarcinoma transition of the tailgut cysts. The patient recovered well and was discharged 10 days after surgery.

**Conclusions:**

We have reported a rare case of retrorectal adenocarcinoma originating from tailgut cysts. MRI, histological, and immunohistological examinations are vital for the diagnosis of tailgut cysts. Complete surgical resection of the tumor should be better performed.

## Background

Tailgut cysts, also known as retrorectal cystic hamartoma, arise from the remnants of the tailgut during the embryonic period [1]. This congenital condition often occurs in middle-aged women and the cysts possess the potential to undergo malignant transformation. Tailgut cysts are located mainly in the retrorectal and anterior sacral spaces, the upper boundary of which forms the peritoneal fold, while the lower boundary is the levator anti, and the bilateral boundary forms the ureters, iliac vessels, and sacral nerve roots [2].

Retrorectal tumors are very rare, occurring with an incidence rate of approximately 1/40,000 [3, 4]. Furthermore, retrorectal tumors originate from a variety of organs and tissues, approximately 60% of which are congenitally residual tissues [3]. Tailgut cysts form in the intestine and malignant transition is rarely seen in the clinic [5]. These cysts are generally asymptomatic and are almost always found during routine physical examinations. As a result, the diagnosis of tailgut cysts is usually delayed. However, patients may present with local effects of the cystic masses, such as the lower abdominal pain, constipation, and rectal tenesmus [2, 6]. These cysts should be carefully monitored for early diagnosis and appropriate treatment, especially when the tailgut cysts are malignant.

Here, we present a very rare case of retrorectal adenocarcinoma originating from tailgut cysts. We also discuss the characteristics, diagnosis, and surgical management of these cysts.

## Case presentation

**Fig. 1:**
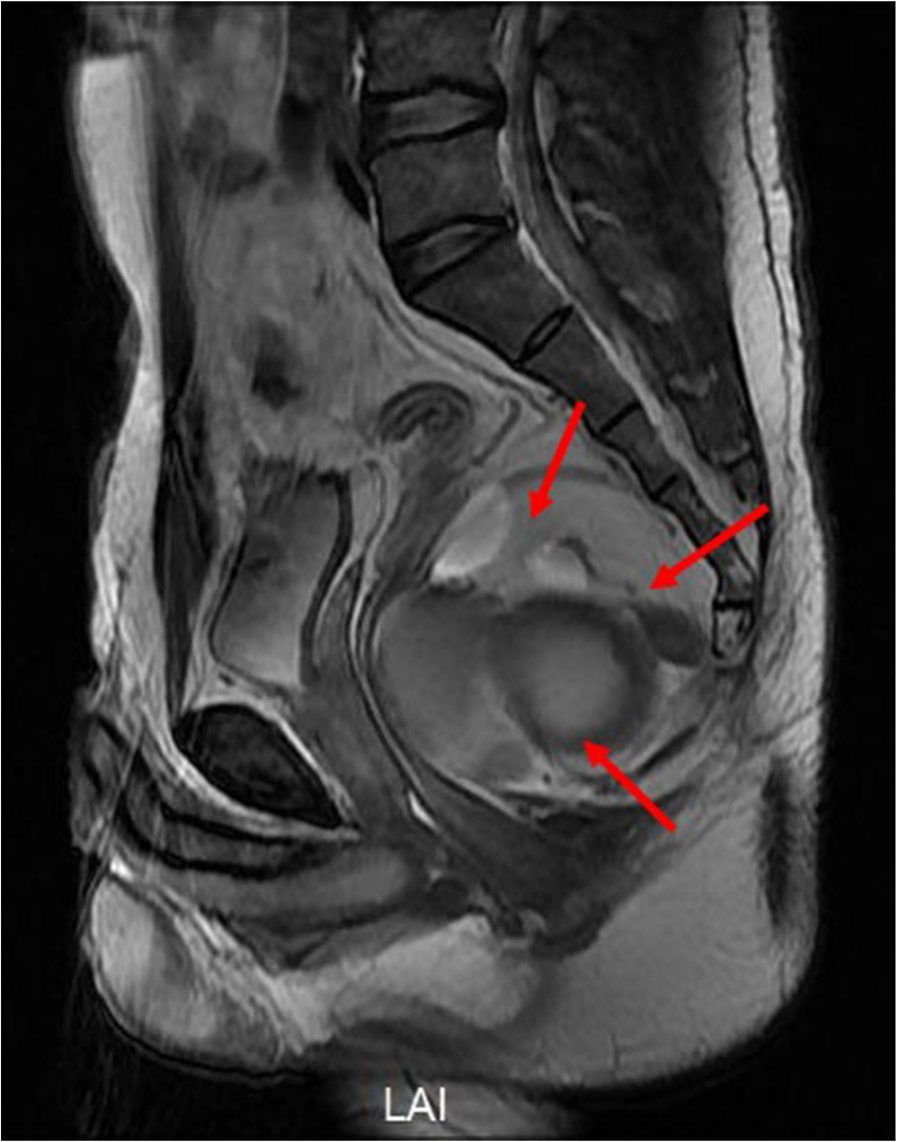
MRI examination of the tailgut cysts. Red arrows indicate the tailgut cysts

A 33-year old man attended our hospital with a sacral mass (80 mm × 59 mm × 53 mm) detected by magnetic resonance imaging (MRI) two days previously. The patient defecated once every 4 to 5 days. The patient had no nausea or vomiting, no abdominal pain or distention, no blood in stools, and no rectal tenesmus. The patient attended our hospital for surgical treatment. During the course of the disease, the general condition, diet, and mental state of the patient were good. There was no history of cough, urinary frequency and urgency, or any other abnormal sensations. Based on the digital rectal examination (knee-chest posture), the mass was palpable within 4 cm at the 12 o’clock position. The mass was solid with a clear boundary, good mobility, and no tenderness. However, the upper edge of the mass could not be felt. In addition, there was no blood or pus staining on the surface of the glove. The MRI examination showed numerous short T1 signals and long T2 signals and many long T1 signals and mixed T2 signals in the posterior rectal space (Fig. 1). The largest mass was approximately 100 mm × 59 mm × 53 mm in size and compressed the rectum and bladder filling was poor. There were no abnormalities in the size of the prostate and bilateral seminal vesicles. There were no enlarged lymph nodes in the bilateral para-femoral and inguinal regions. The MRI examination indicated abnormal signals in the retrorectal and anterior sacral spaces, and the cystic masses were investigated more fully. The carcino-embryonic antigen (CEA) level was 26.97 ng/mL (normal: 0–3 ng/mL) and the carbohydrate antigen 19–9 (CA 19–9) level was 106.50 U/mL (normal: 0–35 ng/mL). However, gastroscopic and enteroscopic examinations showed no signs of gastrointestinal tumor.

**Fig. 2:**
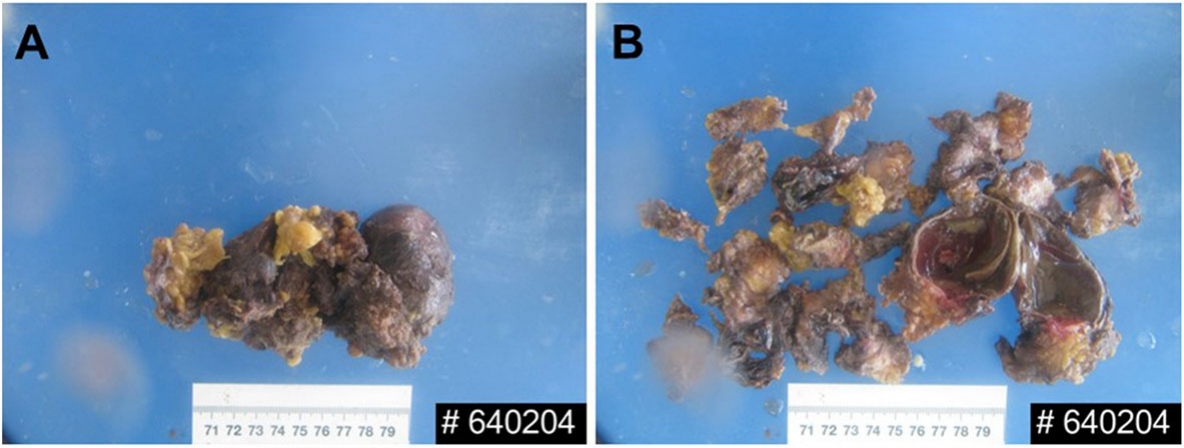
Gross pathology of the tailgut cysts. **a** A 100 mm × 60 mm × 50 mm sized cystic mass. **b** Multiple small tailgut cysts

The patient underwent surgical resection of the masses located in retrorectal and anterior sacral spaces. Lumbar anesthesia was performed with the patient in the left lateral position. An incision of approximately 10 cm was posterior sagittal and located 1 cm to the right of midline. The coccyx tip was removed to expose the presacral mass. During the surgical procedure, a cystic mass was found in the right and posterior lateral region of the rectum and 200 mL pale yellow liquid was drained. The cystic mass was carefully separated and resected. Another mass was observed adjacent to the cystic mass and light green viscous liquid was drained. The mass was also separated and resected (Fig. 2). Complete masses resection with adequate margin was performed to achieve the R0 resection. The resected tissues were sent for further histopathological examination. A drainage tube was placed in the retrorectal and anterior sacral spaces. The operation was performed without problems, and the patient was given oxygen, anti-inflammatories, and fluid replacement treatment.

**Fig. 3:**
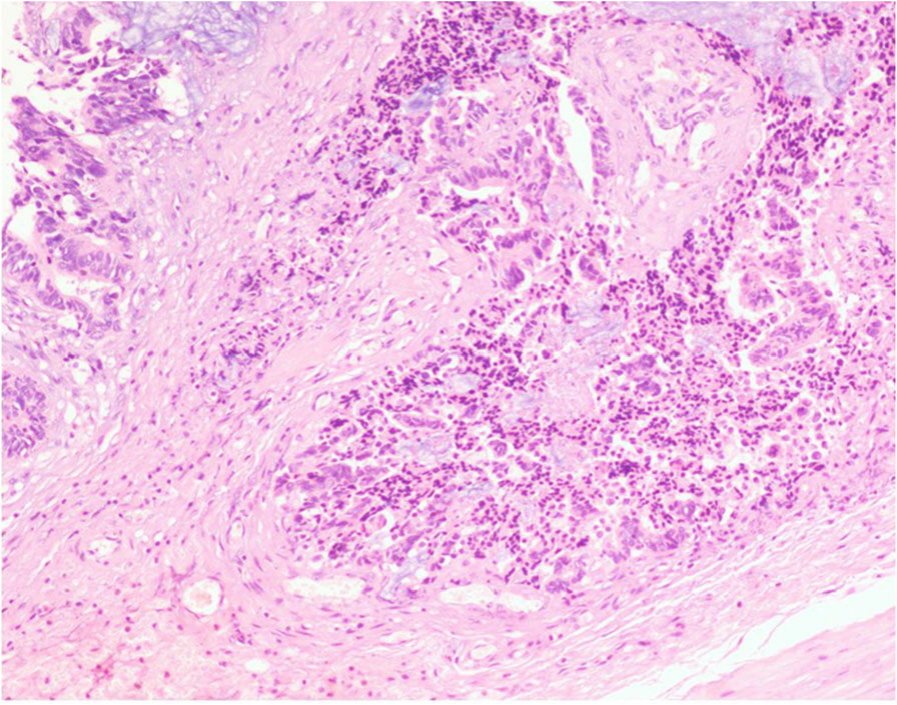
Histological examination (hematoxylin and eosin staining) showed the tailgut cysts accompanied by carcinogenesis. The carcinoma infiltrated the smooth muscle wall and surrounding fibrous tissues

**Fig. 4:**
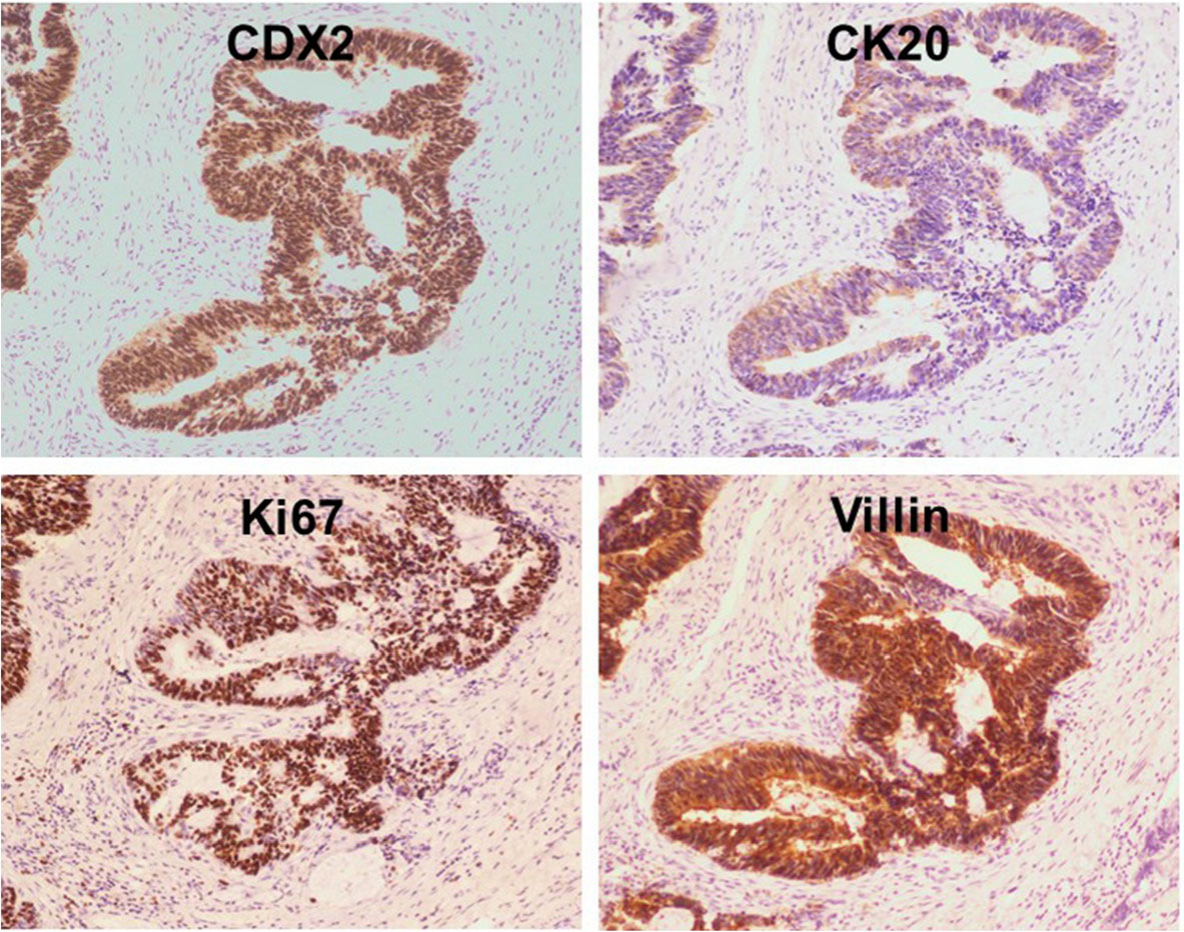
Immunohistological examination of the tailgut cysts

The histopathological examination revealed that the masses were tailgut cysts accompanied by carcinogenesis (high to moderately differentiated adenocarcinoma) infiltrating the smooth muscle wall and surrounding fibrous tissues (Fig. 3). The immunohistological examination showed positive staining of CDX2, CK20, Ki67, and villin, which further indicated the adenocarcinoma transition of the tailgut cysts (Fig. 4). The CEA level was 2.82 ng/mL (normal: 0–3 ng/mL) and the CA 19–9 level was 30.52 U/mL (normal: 0–35 ng/mL) 1 week post-surgery. The patient recovered well and was discharged 10 days post-surgery.

## Discussion and conclusions

Tailgut cysts are a very rare congenital polycystic lesion that always occur in the presacral space, the borders of which are the presacral fascia (Waldeyer fascia) posteriorly, the fascia propria of the rectum and mesorectum anteriorly, levator ani muscles inferiorly, the peritoneal reflection superiorly, and the iliac vessels and ureters laterally [7–10]. The most caudal portion of the hindgut regresses following normal embryogenesis of the anus, rectum, and hindgut and persistence of this embryological remnant in the presacral area is very uncommon [11–14]. Because of the complexity of the anatomy and the embryological development, these cysts were originally classified by Lovelady and Dockerty as either congenital or acquired tumor [15]. The largest study in the literature on tumor development of tailgut cysts was published in 2013 by Sami Akbulut [10].

Prasad et al. proposed that tailgut cysts can be theoretically classified as cystic teratomas [16, 17]. Although malignant transformation of tailgut cysts is very rare, they are generally transitional cell carcinoma, carcinoid tumor, or adenocarcinoma [17]. The patient described here was diagnosed as adenocarcinoma.

In the diagnosis of tailgut cysts, different imaging methods are often faced with numerous differential diagnosis challenges [18]. In the diagnosis of tailgut cysts, computed tomography (CT) and MRI tests are helpful, while the majority of patients with presacral tumors are asymptomatic at presentation. CT provides discrete sectional images of the organs and retroperitoneal compartments. In MRI, tailgut cysts usually have low signal intensity and high signal intensity on T1-weighted images and T2-weighted images, respectively [19, 20]. MRI is more sensitive than CT for differentiation of unilocular and multilocular masses, especially for the detection of small peripheral cysts. Because of the areas of malignant transformation and infection, the cyst wall and septa are usually enhanced by gadolinium [18]. The value and of Tru-Cut biopsy is ambiguous and in this case, this was not recommended due to the risk of tumor-spreading and infection and the possibility of missing the localization. The value of Tru-Cut biopsy is ambiguous and controversial. In this case, biopsy was not recommended due to the risk of tumor-spreading and infection and the possibility of missing the localization. Biopsy can be presented only in unresectable cases or in local, advanced-stage tumors [10].

Surgical management is typically required for nearly all presacral tumors, and preoperative imaging serves as a road map for optimal resection. Although the absolute risk is unclear, some benign presacral tumors may undergo malignant progression, thus necessitating prompt surgical planning once a presacral tumor is diagnosed. Although malignant degeneration/progression may occur, urgent resection of simple, cystic-appearing lesions with features consistent with benign etiologies is unnecessary [21]. However, timely surgical excision should be discussed with the patient, and the risks of pelvic procedures outlined. For tumors the proximal extent of which is below S4, a posterior-only approach is effective (95%) for complete excision, but for those extending above S4, either an abdominal-only or combined abdominal-perineal approach may be best [22]. Whichever approach is adopted, optimal preoperative planning should be based on digital rectal examination, cross-sectional imaging, and pathology, if obtained. During resection, extreme care should be taken not to rupture cystic lesions (or cut across the tumor) to avoid discharge of the contents, which can lead to local recurrences of both benign and malignant tumors. It is worth mentioning that if the patient’s pathological results of preoperative biopsy indicate a malignant disease, it is necessary to resect the biopsy tract during surgery for adequate margin [10].

The spectrum of presacral pathology is broad, but the most commonly seen lesions are developmental cysts, ranging in size from a few centimeters to lesions that completely fill the pelvis [23]. Smaller lesions can be tackled using a transperineal approach [22]. While, very large lesions require an abdominal approach. Malignant degeneration can occur and even benign lesions can adhere intimately to the rectum. As a result, both the patient and surgeon should be prepared for potential proctectomy.

Here, we report a rare case of retrorectal adenocarcinoma originating from tailgut cysts. The diagnosis was confirmed by MRI, histological, and immunohistological examinations. Complete surgical resection of the tumor was performed, and the patient recovered well. We also describe the characteristics, diagnosis, and surgical management of retrorectal tumors.

## Data Availability

The datasets supporting the conclusions of this article is included within the article.

## Abbreviations

CA 19–9: Carbohydrate antigen 19–9
CEA: Carcino-embryonic antigen
CT: Computed tomography.
MRI: Magnetic resonance imaging
